# Protocol for the derivation and validation of a clinical prediction model to support the diagnosis of asthma in children and young people in primary care

**DOI:** 10.12688/wellcomeopenres.15751.1

**Published:** 2020-03-24

**Authors:** Luke Daines, Laura J. Bonnett, Andy Boyd, Steve Turner, Steff Lewis, Aziz Sheikh, Hilary Pinnock

**Affiliations:** 1 Asthma UK Centre for Applied Research, Usher Institute, University of Edinburgh, Edinburgh, EH8 9AG, UK; 2 Department of Biostatistics, University of Liverpool, Liverpool, UK; 3 Institute of Population Health Science, University of Bristol, Bristol, UK; 4 Department of Child Health, University of Aberdeen, Aberdeen, UK

**Keywords:** Asthma, Diagnosis, Primary Care, Clinical Prediction Models, ALSPAC

## Abstract

**Background: **Accurately diagnosing asthma can be challenging. Uncertainty about the best combination of clinical features and investigations for asthma diagnosis is reflected in conflicting recommendations from international guidelines. One solution could be a clinical prediction model to support health professionals estimate the probability of an asthma diagnosis. However, systematic review evidence identifies that existing models for asthma diagnosis are at high risk of bias and unsuitable for clinical use. Being mindful of previous limitations, this protocol describes plans to derive and validate a prediction model for use by healthcare professionals to aid diagnostic decision making during assessment of a child or young person with symptoms suggestive of asthma in primary care.

**Methods:** A prediction model will be derived using data from the Avon Longitudinal Study of Parents and Children (ALSPAC) and linked primary care electronic health records (EHR). Data will be included from study participants up to 25 years of age where permissions exist to use their linked EHR. Participants will be identified as having asthma if they received at least three prescriptions for an inhaled corticosteroid within a one-year period and have an asthma code in their EHR. To deal with missing data we will consider conducting a complete case analysis. However, if the exclusion of cases with missing data substantially reduces the total sample size, multiple imputation will be used. A multivariable logistic regression model will be fitted with backward stepwise selection of candidate predictors.  Apparent model performance will be assessed before internal validation using bootstrapping techniques. The model will be adjusted for optimism before external validation in a dataset created from the Optimum Patient Care Research Database.

**Discussion: **This protocol describes a robust strategy for the derivation and validation of a prediction model to support the diagnosis of asthma in children and young people in primary care.

## Introduction

Asthma affects an estimated 339 million people worldwide ^1^ but is commonly mis-diagnosed in children and adults in primary care ^2, 3^. Incorrectly labelling an individual with asthma can result in the prescription of inappropriate treatment and the underlying cause of symptoms being missed. On the other hand, not recognising that an individual has asthma can lead to ongoing symptoms, reduced quality of life and risk of asthma attack.

In primary care, a diagnosis of asthma is commonly based on clinical features, previous history together with evidence of variable airflow limitation or, more recently, airway inflammation ^4, 5^. However, as a heterogeneous and variable condition, the clinical features of asthma can differ according to phenotype, and due to the varying intensity of symptoms and signs over time ^5, 6^. In addition, no single investigation can confirm or refute asthma in every situation ^4^. Consequently, the best strategy for confirming an asthma diagnosis in primary care remains unclear ^7– 9^, and national/international guidelines recommend conflicting diagnostic strategies ^4, 5, 10^, which has led to confusion and uncertainty amongst health professionals ^11^.

A clinical prediction model for asthma diagnosis in primary care could support diagnostic decision making by providing the probability that asthma is present using information from clinical symptoms, signs, medical and family history or tests. A recent systematic review identified seven clinical prediction models for asthma diagnosis in primary care, six models were derived in adults, and one in children ^12^. Unfortunately each of the prediction models had methodological limitations and were considered unsuitable for clinical practice ^12^. Therefore, this protocol describes plans to derive and internally and externally validate a prediction model intended for use by a primary healthcare professional to aid their diagnostic decision making during the assessment of a child or young person with symptoms suggestive of asthma.

## Methods

### Source of data: derivation and internal validation

We will derive a clinical prediction model using participant-reported data from the Avon Longitudinal Study of Parents and Children (ALSPAC) and participant’s linked primary care electronic health records (EHR). ALSPAC is a prospective observational study that recruited pregnant women resident in and around the City of Bristol, UK with expected dates of delivery 1st April 1991 to 31st December 1992. The initial (recruitment Phase I) number of pregnancies enrolled was 14,541 (14,676 foetuses, resulting in 14,062 live births and 13,988 children who were alive at 1 year of age). When the children from Phase I were approximately seven years of age, attempts were made to bolster the recruited sample with eligible cases who had not joined the study originally. By the time children from Phase I were 24 years of age, a further 913 index children had been recruited (456, 262 and 195 recruited during Phases II, III and IV, respectively) ^13– 15^. The total recruited sample available for analyses is therefore 15,454 pregnancies, resulting in 15,589 foetuses. Of these 14,901 were alive at one year of age. Frequent assessments of participants have been conducted with 68 data collection points between birth and 18 years of age ^13^. At Phase I enrolment, 49.7% of children were female and 96.1% were of White ethnicity ^13^. The ALSPAC website ( www.bristol.ac.uk/alspac/) contains details of all available data through a searchable dictionary and variable search tool. REDCap electronic data capture tools have been used to collect and manage ALSPAC study data since 2014 ^16, 17^. To enhance the ALSPAC resource, the Project to Enhance ALSPAC through Record Linkage (PEARL) has established the requisite ethico-legal permissions to link to and extract participant’s primary care EHR, allowing data from clinical consultations and prescribing to be utilised. When the index children reached legal adulthood (i.e. age 18), ALSPAC conducted a postal fair processing campaign which aimed to re-enrol them into the study in their own right and to inform participants about the planned incorporation of linked health and administrative records in the ALSPAC databank. Participants had the right to opt-out of this use of their information (applied both at a study-level and in accordance with the UK’s National Opt-Out mechanisms). ALSPAC subsequently sought permission from general practitioner (GP) practices with registered ALSPAC participants to extract linked records. Participants were identified in the GP record using National Health Service (NHS) ID number and extracted by practice system software companies. Linkage to GP records was carried out following this campaign. Only coded data from the primary care records were extracted.

### Participants

Study participants will be chosen from the ALSPAC dataset using pre-specified eligibility criteria. For the derivation dataset, the inclusion criteria will be participants:

- recruited into the original cohort (i.e. at Phase I, because children recruited at a later stage have missing data from birth to seven years of age)
- alive at one year
- with consent for the use of their linked EHR

### Outcome

The outcome measure will be derived from linked primary care EHR. We will use prescribing data in combination with the presence of an asthma Read code (version 2) to identify the presence of asthma and the date of diagnosis. We will identify participants who received at least three prescriptions for an inhaled corticosteroid, as a single inhaler or in combination with a long-acting beta agonist, on separate days within a one-year period. From this group, we will select participants who have an asthma ‘specific’ Read code (according to the validated code list from Nissen *et al.*) ^18^ occurring at any time in their patient record. Individuals with at least three inhaled corticosteroid prescriptions in one year and a ‘specific’ asthma Read code will be designated as having asthma (see *Extended data* ^19^ for the Read code lists to be used in this study).

This prediction model is intended for use by health professionals at the point of asthma diagnosis. It is therefore important to ensure that any information that occurs after the diagnosis is excluded from the development dataset. To do this, an event date is required. The event date for those with the outcome will be taken as the date at which the first of the inhaled corticosteroid prescriptions was made. Those without the outcome have no equivalent event date. Therefore, participants without an outcome will be assigned an event date at random. To do so participants with an outcome will be grouped by their age (years) at event date and the proportion of individuals within each year age group will be taken. Then participants without an outcome will be randomly sorted into age-at-event groups so that the same proportion of individuals will be placed in the age-at-event groups. Other than age-at-event, (which will no longer be available for modelling), the outcome measure will be developed blind to information about the predictors. 

### Predictors

Potential candidate predictors were identified based on the results from our systematic review of prediction models for the diagnosis of asthma in primary care ^12^ and based on clinical usefulness decided after discussion within the research team (including GPs, respiratory paediatricians and statisticians). We will choose the final list of candidate predictors from the following: gender, social class, wheeze, cough, night cough, breathlessness, eczema, hay fever, allergy to food or drink, allergy to substance other than food or drink, maternal asthma, maternal atopy, maternal cigarette smoking during pregnancy, childhood exposure to cigarette smoke, mould in the participants house, lung function indices from spirometry, fractional exhaled nitric oxide (FeNO), skin prick testing results, immunoglobulin E (IgE) serum samples, evidence of lung function or reversibility testing in the patient EHR and prescription of a short-acting beta agonist (SABA).

### Sample size

The number of candidate predictor variables will be restricted to a minimum of 10 events per variable. Following preliminary analysis of the ALSPAC dataset, 11972 participants met the eligibility criteria, with 994 participants having the outcome of interest. Taking into account the 22 candidate predictors (27 parameter levels) and the number of outcome events, the events per variable (36.8) far exceeds recommendations for sample sizes ^20^.

### Missing data

Different approaches to dealing with missing data will be considered. Variables with over 40% missingness will be excluded. Given the large number of participants, conducting a complete case analysis will be considered by running estimations using the full list of candidate predictors. If the exclusion of cases with missing data substantially reduces the total sample size, then multiple imputation by creating up to 20 imputed datasets via chained equations ^21^ will be considered ^22^.

### Statistical analysis methods

Statistical analysis will be conducted using R (version 3.5.3), and SPSS (version 26).

***Handling of predictor variables.*** Where possible, variables will be used in the form in which they were collected – for example, a continuous variable will not be split into categories unless necessary ^23^. Continuous variables will be checked for linearity and if necessary, fractional polynomials used to improve the fit of non-linear relationships ^24^.

Variables relating to participant characteristics and symptoms were captured in the ALSPAC study by child and parent completed questionnaires. As questionnaires were completed on a number of occasions, many symptoms/exposures (such as wheeze) were collected on more than one occasion. Fortunately, ALSPAC used the same (or very similar) questions making it possible to combine responses collected at different time points. When computing variables into candidate predictors these data will initially be considered as counts; describing the number of times a symptom/exposure occurred before the event date. However, if there are a large proportion of zeros, we will recode the variable into a binary category, thus capturing the presence or absence of a symptom/exposure prior to the event date.

Two candidate predictors will be derived from linked EHR. To capture information relating to the evidence of lung function or reversibility testing, a list of relevant codes was compiled. The occurrence of any lung function or reversibility Read code (see *Extended data*) ^19^ will be extracted from the linked EHR together with an anonymised identifier and event date. From these data a binary variable will be created describing the presence or absence of a ‘lung function/reversibility’ code occurring before the event date. A binary variable identifying the presence or absence of a SABA prescription prior to the event date will be similarly constructed, though the code list will be compiled from the Systematized Nomenclature of Medicine (SNOMED) prescribing terms ^25^.

***Type of model.*** We intend to use multivariable logistic regression as this is an appropriate method where outcome measures are binary and candidate predictor variables are categorical, continuous, or combined ^26, 27^.

***Predictor selection before modelling.*** From the list of candidate predictors, predictors missing in more than 40% of participants will be excluded. Where inter-relatedness between predictors exists, the predictors that best capture the information sought will be retained by choosing firstly based on clinical relevance, and secondly (if variables are equally relevant), the predictor with least missing data. We will not use univariate analysis to screen for significant associations between potential predictors and the outcome, as predictors behave differently in a multivariable model ^28^.

***Predictor selection during modelling.*** Backward step-wise selection based on the Akaike’s Information Criterion (AIC) will be used to select predictor variables during modelling ^29^.

***Model performance.*** The apparent performance of the final model will be calculated in the original sample. Discrimination will be reported using the concordance statistic (c-statistic). Calibration will be assessed visually using a calibration plot and by calculating the calibration slope, calibration-in-the-large (CITL) and ratio of expected and observed number of events (E/O) ^30^.

### Internal validation

Rather than reduce the sample size by using split-sample or cross-validation, bootstrapping techniques for model validation will be used ^30^. The modelling process including variable selection will be repeated in up to 500 samples drawn with replacements from the original sample. The bootstrap performance of the model will be assessed in each bootstrap sample using the c-statistic. We will determine the performance of the bootstrap model in the original sample (test performance) and calculate the optimism as the difference between the bootstrap and test performance. We will average the estimates of optimism from each bootstrap sample and subtract the value from the apparent performance to generate an optimism-corrected estimate of performance. The optimism adjusted calibration slope will be used as the shrinkage factor to adjust the regression coefficients of the developed model for optimism.

### External validation

External validation of the model will be conducted in a dataset created from routinely collected anonymised primary care records from the Optimum Patient Care Research Database. Using the model derived in the ALSPAC dataset (and adjusted for shrinkage), we will calculate the linear predictor and predicted probability of the outcome for each individual in the external validation dataset. Model performance will be assessed using measures of discrimination (c-index) and calibration (calibration plot, calibration slope, CITL and E/O) ^30^. Recalibration of the model will be completed if there is a systematic under- or over-prediction of risk in the external validation dataset.

## Risk groups

The potential for generating risk groups using the probabilities from the multivariable model will be considered. Currently, the British Thoracic Society/Scottish Intercollegiate Guideline Network recommend that clinicians weigh up the probability of an asthma diagnosis into high, intermediate and low probability ^4^. Therefore, defining the probability generated from the prediction model in such terms may appeal to clinicians. However, the range of predicted probabilities incorporated within a group, and the benefit of constructing risk groups will require further consideration and the involvement of patients and clinicians ^31^.

## Discussion

Current clinical prediction models for the diagnosis of asthma are at high risk of bias and not recommended for use in clinical practice ^12^. This protocol builds on the findings from our systematic review to derive and validate a clinical prediction model for primary healthcare professionals to support their decision making during the assessment of a child or young person with symptoms to suggest asthma. The protocol has been guided by the Transparent Reporting of a multivariable prediction model for Individual Prognosis Or Diagnosis (TRIPOD) ^26^. We intend to include a broad range of predictor variables, including demographics, symptoms, medical and family history, and ideally results from clinical tests. We have a robust plan to deal with missing data, assess model performance, internally validate the model and take account of model optimism.

ALSPAC has only been given permissions to extract coded information from the medical records due to concerns that free-text notes may pose a confidentiality risk (relating to third-parties rather than the participant themselves). This means the prediction model development will only make use of coded information, and thus cannot take advantage of the increase in algorithm sensitivity that using free-text brings ^32^. The use of coded diagnosis in combination with prescription records mitigates the risk of ‘false positive’ identification of cases resulting from GPs recording practice where a code is entered for a tentative diagnosis or a coded diagnosis entry is added to the record along with free-text information indicating the patient does not have the condition ^33^.

Following the derivation and internal validation of a clinical prediction model in the ALSPAC dataset, we will externally validate the model in routinely collected data and explore the implementation of the model into clinical practice.

## Declarations

### Ethics approval and consent to participate

Ethical approval for the derivation and internal validation was obtained from the ALSPAC Ethics and Law Committee and the Local Research Ethics Committees. ALSPAC’s use of linked health records is based on approvals from the ALSPAC Ethics and Law Committee, Health Research Authority Research Ethics Committee and Confidentiality Advisory Group. Informed consent for the use of data collected via questionnaires and clinics was obtained from participants following the recommendations of the ALSPAC Ethics and Law Committee at the time. Ethical approval to complete external validation of a clinical prediction model for the diagnosis of asthma in primary care was obtained from the Anonymised Data Ethics and Protocol Transparency Committee (ADEPT) (Approval Reference: ADEPT0320).

### Consent for publication

ALSPAC participants have been provided with fair processing materials describing the studies use of the data they have provided or those collected through record linkage and about the legal basis under which the study operates: this includes the sharing of de-identified data with researchers and the publishing of research findings. Study members have the right to withdraw from elements of the study or from the study entirely at any time. Full details of the ALSPAC consent procedures are available from the study website.

## Data availability

### Underlying data

No underlying data are associated with this article.

ALSPAC data access is through a system of managed open access. The steps below highlight how to apply for access to the data referred to in this article and all other ALSPAC data. The datasets presented in this article are linked to ALSPAC project number B2830, please quote this project number during your application. The ALSPAC variable codes highlighted in the dataset descriptions can be used to specify required variables.

- 1. Please read the ALSPAC access policy ( https://www.bristol.ac.uk/media-library/sites/alspac/documents/researchers/data-access/ALSPAC_Access_Policy.pdf) which describes the process of accessing the data and samples in detail, and outlines the costs associated with doing so.
- 2. You may also find it useful to browse our fully searchable research proposals database ( https://proposals.epi.bristol.ac.uk/), which lists all research projects that have been approved since April 2011.
- 3. Please submit your research proposal for consideration by the ALSPAC Executive Committee. You will receive a response within 10 working days to advise you whether your proposal has been approved.

If you have any questions about accessing data, please email alspac-data@bristol.ac.uk.

The study website also contains details of all the data that is available through a fully searchable data dictionary: http://www.bristol.ac.uk/alspac/researchers/data-access/data-dictionary/.

### Extended data

Open Science Framework: Clinical prediction model for the diagnosis of asthma in children and young people in primary care. https://doi.org/10.17605/OSF.IO/FU4GN ^19^.

This project contains the following extended data:

- AsthmaSpecific_ReadcodeList (TXT). (Asthma-specific read codes.)
- LungFunctionAndReversibility_ReadCodeList (TXT). (Lung function/reversibility testing read codes.)

Extended data are available under the terms of the Creative Commons Attribution 4.0 International license (CC-BY 4.0).
