# Supplementary material for: Protocol for the derivation and validation of a clinical prediction model to support the diagnosis of asthma in children and young people in primary care
Source: Wellcome Open Res. 2020 Mar 24;5:50. [Version 1] doi: 10.12688/wellcomeopenres.15751.1 (PMC7364181; doi:10.12688/wellcomeopenres.15751.1)
Supplement: Peer review history

# Peer review: Reviewer response for version 1

**DOI:** 10.21956/wellcomeopenres.17271.r39328

**Published:** 2020-07-15

**Authors:** Patrick J E Bindels

**Affiliations:** 1 Department of General Practice, Erasmus MC, Rotterdam, The Netherlands

This is a protocol for the derivation and validation of a clinical prediction model to support the diagnosis of asthma in children and young people. The prediction model will be developed for use in primary care. As the authors stated the diagnosis of asthma in primary care is based predominantly on clinical features in combination with demographics, medical history and ideally results from clinical tests. It would be of great help for general practitioners if a prediction model could assist them in classifying children in low, intermediate and high risk of developing asthma. Especially in the very young children (< 6 years) a probable diagnosis of asthma is a challenge in general practice.

 In this protocol, the researchers are going to derive a model from the ALSPAC study in combination with data form the primary care EHR of the participants in the ALSPAC study. External validation is planned in the Optimum Patient Care Research Database.

 It is an interesting protocol and any attempt to develop a prediction model for general practice is highly relevant. I do have some comments and I hope these comments will help to improve the protocol further.

- Especially in pre-school children, a presumptive asthma diagnosis is a true challenge for general practitioners. Clinical features are not always very specific and the more ‘classical’ symptoms like shortness of breath (or the presence of inhalation allergy) not present. From the age of 6 onwards, the clinical features of asthma-like shortness of breath and wheeze are more explicit. It makes a diagnosis of asthma in older children less a challenge for most GP’s (a diagnosis is often not the problem anymore, undertreatment however is). Moreover, from the age of 5-6 years, lung function testing is possible in children. Besides clinical symptoms, a more objective measurement (FEV1 and reversibility testing) is available to support an asthma diagnosis. Will it be possible to construct an overall prediction model for young children and young people? Or will it be necessary to construct prediction models for specific age groups (e.g. < 6 years, 6-12 years, and adolescents) where each age specific prediction model will have its own specific variables?
- In the majority of patients, allergic asthma starts at a young age and persists during adolescence and adulthood. A first clinical presentation of asthma at adolescence (without any symptoms as a child) is to my knowledge rare. I am sure the authors do have a specific reason for also developing this prediction score for young people?
- Clinical symptoms will probably be important variables in the prediction model (wheeze, cough, night cough, breathlessness). The variables will be captured form self-reported questionnaires by the parent or child. It is well known that there can be a considerable difference in self-reported symptoms (in questionnaires) and symptoms registered in an EHR by a GP. Especially for wheeze. Is the information on the validity of the self-reported symptoms in ALSPAC available?
- When computing variables into candidate predictors these data will be considered as counts (responses at different time points will be combined). The number of times a symptom occurred before the event date will be used as a candidate variable. However, a child will not have visited the GP each time a period of cough or wheeze occurred. Let’s presume having had three periods of wheeze in the last year is a variable in the final model. The information is based on questionnaires data and not on clinical presentation. Can a GP rely on self-counted episodes of symptoms by the parent, especially in very young children?
- Did the authors consider starting the follow up of patients at the first possible clinical presentation of asthma (preferably the first presentation in a clinical setting because that is the moment a GP might start to consider a possible diagnosis of asthma)?   
- Two candidate predictors will be derived from the linked EHR (lung function and SABA prescriptions). In the list of potential candidate predictors also skin prick testing, IgE en FeNO are considered (but not through linkage with the EHR). Are these variables available in ALSPAC?
- The outcome measure will be derived from prescribing data in combination with the presence of an asthma Read code. The authors will only identify participants who received at least three prescriptions for an inhaled corticosteroid (within one year). This might mean a selection of more severe asthma (or children who are therapy compliant!) and children with mild asthma (one or two prescriptions in combination with SABA prescriptions or children who are not compliant but do have asthma) will be excluded. Compliance will be a problem that has to be taken into account especially in adolescents?
- External validation of the model will be conducted in the Optimum Patient Care Research Database. The information on this database is limited in the protocol. What kind of database is it? An EHR based database? How are symptoms (and other potential variables) registered in this database? Is it done in the same way as in ALSPAC? For instance, in ALSPAC symptoms are based on self-reported questionnaires, how are symptoms registered in Optimum?
- It is an interesting protocol and I am looking forward to the model that will be derived.

Is the study design appropriate for the research question?

Yes

Is the rationale for, and objectives of, the study clearly described?

Yes

Are sufficient details of the methods provided to allow replication by others?

Partly

Are the datasets clearly presented in a useable and accessible format?

Yes

Reviewer Expertise:

Research in general practice

I confirm that I have read this submission and believe that I have an appropriate level of expertise to confirm that it is of an acceptable scientific standard.

# Peer review: Reviewer response for version 1

**DOI:** 10.21956/wellcomeopenres.17271.r39329

**Published:** 2020-07-02

**Authors:** Glen Martin

**Affiliations:** 1 Division of Informatics, Imaging and Data Science, Faculty of Biology, Medicine and Health, Manchester Academic Health Science Centre, University of Manchester, Manchester, UK

In this study protocol, the authors describe the plans for developing and validating (both internally and externally) a prediction model that will aim to estimate the risk of an asthma diagnosis. To develop and internally validate the model, the authors plan to use data from the Avon Longitudinal Study of Parents and Children (ALSPAC), which will be linked with primary care data. External validation will be based on a dataset created from the Optimum Patient Care Research Database.

 The motivation of the work is clear, based on a previous systematic review, which identified that previous models in this area are at high risk of bias, and have not been deemed suitable for clinical practice.

 From a statistical viewpoint, I think that the protocol is very clear and includes a detailed description of the methods that will be used. It was good to see that the protocol (and subsequent study) follows the TRIPOD guidelines. The methodology that will be used to develop the prediction model is sound and considers important aspects such as correctly handling continuous variables (including consideration of non-linear associations), predictor/ outcome definitions, and handling of missing data.

 I have several comments, which the authors might like to consider and are intended to be constructive in helping to improve the protocol further. I list them here in the order that they occurred to me when reading the protocol:

1. I found the description of the outcomes slightly unclear, particularly the first paragraph of the subsection. The authors say that they will use “three prescriptions for an inhaled corticosteroid” and “the presence of an asthma Read code” to define the outcome. What motivated the choice of using three prescriptions of an inhaled corticosteroid in a single year? Is this the standard way of defining an asthma diagnosis in routine data? As I understand from the text, the authors will only look for the asthma Read codes in the subgroup identified as having the three prescriptions (i.e. anyone with an asthma Read code but who doesn’t have at least three prescriptions for an inhaled corticosteroid in a single year will be defined as not having asthma). While I think that this would reduce any false positives of the outcome definition, it might help the protocol if the authors justified the strict use of three prescriptions in a single year (since this could increase false negatives).
2. In the Sample size subsection, the authors justify the sample size based on events per variable (using the commonly applied EPV>10 rule-of-thumb). The justification for the sample size could be further improved if the authors also applied recent formal sample size criteria by Riley *et al.* (e.g. https://doi.org/10.1002/sim.7992 for logistic models) ^1^. These criteria would allow the authors to directly show that the sample size is sufficient to minimise overfitting, for a given number of candidate predictors. These criteria require pre-specification of the anticipated Cox-Snell R2, so the authors could use their previous systematic review of existing asthma diagnosis risk models to do this. I suspect, given the relatively large sample size (EPV of 36.8), that the data will exceed these calculations, but determining this a priori would be a valuable addition to the protocol, in my view.
3. I valued the plans to use both clinical insights and data-driven methods to guide predictor selection. I just have one minor suggestion here: rather than using backward stepwise selection based on AIC for predictor selection during modelling, I wonder if using LASSO would be beneficial since this can select variables, while also penalising/shrinking parameter estimates. The latter characteristic would further minimise the risk of overfitting to the development data.
4. Model performance subsection: How will the calibration plots be constructed: will this be based on the decile method or based on a flexible calibration plot ( https://pubmed.ncbi.nlm.nih.gov/26772608/) ^2^?
5. Internal validation: “The bootstrap performance of the model will be assessed in each bootstrap sample using the c-statistic” – presumably, the end of this sentence should read “…using the c-statistic, calibration intercept and calibration slope”, since one needs to estimate the optimism in all of the performance measures? I think this is simply an omission of text since the authors refer to the “optimism adjusted calibration slope” later in the paragraph.
6. External validation: are all the variables that are available in the ALSPAC dataset (linked with EHR) and will be considered for inclusion in the model also available in the Optimum Patient Care Research Database that will be used for external validation? If not, how will the authors handle the situation where a variable in the developed model is not in the external validation dataset? Either way, it might be useful to add some additional detail of the Optimum Patient Care Research Database, including how the authors will define the cohort used for external validation (which will presumably be the same as that described for model development/ internal validation).

Overall, a great protocol for a study that has clear motivation. I hope my comments are useful in refining the protocol further.

Is the study design appropriate for the research question?

Yes

Is the rationale for, and objectives of, the study clearly described?

Yes

Are sufficient details of the methods provided to allow replication by others?

Yes

Are the datasets clearly presented in a useable and accessible format?

Yes

Reviewer Expertise:

Statistics/ Risk prediction modelling/ Prognostic research

I confirm that I have read this submission and believe that I have an appropriate level of expertise to confirm that it is of an acceptable scientific standard.

## References

[1] : Minimum sample size for developing a multivariable prediction model: PART II - binary and time-to-event outcomes. *Statistics in Medicine*.2019;38(7) : 10.1002/sim.7992 1276-1296 10.1002/sim.7992 PMC651926630357870

[2] : A calibration hierarchy for risk models was defined: from utopia to empirical data. *J Clin Epidemiol*.74: 10.1016/j.jclinepi.2015.12.005 167-76 10.1016/j.jclinepi.2015.12.005 26772608
